# Evaluation of an open-source pipeline to create patient-specific left atrial models: A reproducibility study

**DOI:** 10.1016/j.compbiomed.2023.107009

**Published:** 2023-08

**Authors:** José Alonso Solís-Lemus, Tiffany Baptiste, Rosie Barrows, Charles Sillett, Ali Gharaviri, Giulia Raffaele, Orod Razeghi, Marina Strocchi, Iain Sim, Irum Kotadia, Neil Bodagh, Daniel O’Hare, Mark O’Neill, Steven E. Williams, Caroline Roney, Steven Niederer

**Affiliations:** a School of Biomedical Engineering & Imaging Sciences, King’s College London, St Thomas Hospital, London, SE1 7EH, UK; b Queen Mary University of London, Mile End Rd, Bethnal Green, London, E1 4NS, UK; c Centre for Cardiovascular Science, University of Edinburgh, Old College, South Bridge, Edinburgh, EH8 9YL, Scotland, UK; d Department of Haematology, NHS Blood and Transplant Centre, University of Cambridge, Cambridge, UK; e School of Medical Education, King’s College London, St Thomas Hospital, London, SE1 7EH, UK; f Alan Turing Institute, British Library, 96 Euston Rd, London, NW1 2DB, UK

**Keywords:** Reproducibility, Atrial imaging, Patient-specific modelling, Cardiac electrophysiology, Image analysis, Digital twins

## Abstract

This work presents an open-source software pipeline to create patient-specific left atrial models with fibre orientations and a fibrDEFAULTosis map, suitable for electrophysiology simulations, and quantifies the intra and inter observer reproducibility of the model creation. The semi-automatic pipeline takes as input a contrast enhanced magnetic resonance angiogram, and a late gadolinium enhanced (LGE) contrast magnetic resonance (CMR). Five operators were allocated 20 cases each from a set of 50 CMR datasets to create a total of 100 models to evaluate inter and intra-operator variability. Each output model consisted of: (1) a labelled surface mesh open at the pulmonary veins and mitral valve, (2) fibre orientations mapped from a diffusion tensor MRI (DTMRI) human atlas, (3) fibrosis map extracted from the LGE-CMR scan, and (4) simulation of local activation time (LAT) and phase singularity (PS) mapping. Reproducibility in our pipeline was evaluated by comparing agreement in shape of the output meshes, fibrosis distribution in the left atrial body, and fibre orientations. Reproducibility in simulations outputs was evaluated in the LAT maps by comparing the total activation times, and the mean conduction velocity (CV). PS maps were compared with the structural similarity index measure (SSIM). The users processed in total 60 cases for inter and 40 cases for intra-operator variability. Our workflow allows a single model to be created in 16.72 ± 12.25 min. Similarity was measured with shape, percentage of fibres oriented in the same direction, and intra-class correlation coefficient (ICC) for the fibrosis calculation. Shape differed noticeably only with users’ selection of the mitral valve and the length of the pulmonary veins from the ostia to the distal end; fibrosis agreement was high, with ICC of 0.909 (inter) and 0.999 (intra); fibre orientation agreement was high with 60.63% (inter) and 71.77% (intra). The LAT showed good agreement, where the median ± IQR of the absolute difference of the total activation times was 2.02 ± 2.45 ms for inter, and 1.37 ± 2.45 ms for intra. Also, the average ± sd of the mean CV difference was -0.00404 ± 0.0155 m/s for inter, and 0.0021 ± 0.0115 m/s for intra. Finally, the PS maps showed a moderately good agreement in SSIM for inter and intra, where the mean ± sd SSIM for inter and intra were 0.648 ± 0.21 and 0.608 ± 0.15, respectively. Although we found notable differences in the models, as a consequence of user input, our tests show that the uncertainty caused by both inter and intra-operator variability is comparable with uncertainty due to estimated fibres, and image resolution accuracy of segmentation tools.

## Highlights

• New open-source pipeline creates patient-specific left atrial models from CMR scans.
• Inter/intra-operator variability evaluated in study with 100 models.
• Model building time reduced to just 16.7 min.
• Error measurements from operator variability comparable to differences observed with varied Image resolution or fibre field.

## Graphical abstract

**Figure:**
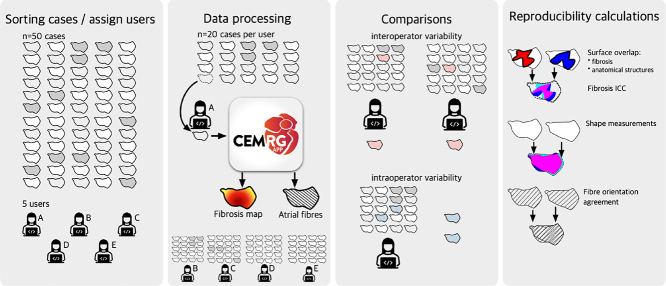

## 1 Introduction

Patient-specific computational models of the heart are moving from a research tool to industrial and clinical applications [1]. Regulatory bodies and societies are now providing verification, validation and uncertainty quantification frameworks for the evaluation of these models, and the steps and tests required to demonstrate model credibility for their context of use [2].

In the area of patient-specific cardiac models, previous research has addressed areas of code verification providing N-version and analytical benchmark problems [3], [4], [5], availability of independent validation data sets, and adoption of uncertainty quantification techniques [6]. However, little attention has been given to the uncertainty introduced by operator decisions in patient-specific cardiac modelling workflows. In particular, the impact of **intra-operator variability**, which refers to the variation in measurements made by the same operator when performing a task multiple times, and **inter-operator variability**, which refers to the variation in measurements made by different operators when performing the same task, has not been adequately addressed. This knowledge gap is particularly relevant considering the routine reporting of intra and inter-observer variability in medical imaging [7], [8].

**Fig. 1:**
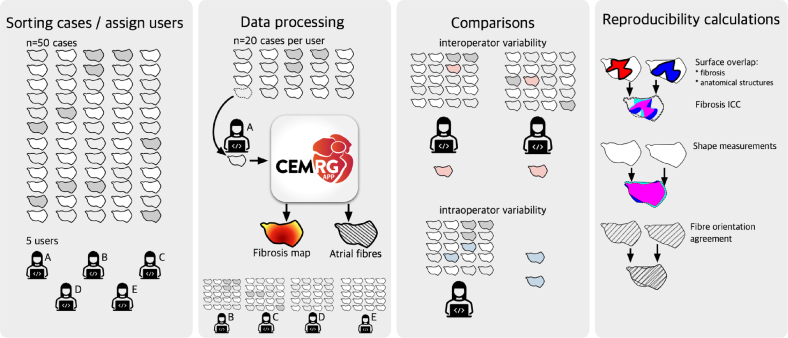
Overview of this study. Five independent users processed 20 cases at random from a pool of 50 cases. Processing was done using the CemrgApp pipeline developed for this work, which involves the creation of a simulation-ready mesh, fibres orientations, and a fibrosis map. Users processed some cases twice to test for intra-operator variability, whilst other cases were processed by two users to test for inter-operator variability. The pipeline was assessed for reproducibility at various stages: the surface area overlap, shortest distance of each points, and fibre orientations. Each of the 100 output cases (20 × 5 users) were used to run 3 electrophysiology simulations, where total activation time, absolute LAT differences, and correlation of PS map in universal atrial coordinates (UAC) were calculated.

The high cost in model creation to date and manual workflows have limited the evaluation of model creation reproducibility. Performing inter and intra-observer variability studies is bound by the capacity to analyse sufficiently large data sets by different operators. However, the capacity to create such data sets is currently limited, as past modelling studies have built only a few patient-specific models, often fewer than 10. While more recent studies have included 20 to 50 patients [9], only a few studies with 80 or more cases have been reported [10], [11]. To enable and motivate studies on intra and inter-observer variability, it is necessary to develop robust software platforms that facilitate the creation of larger cohorts of models.

CemrgApp [12] is an open-source platform, aggregating different open source workflows, for performing medical image analysis and creating patient-specific models. Specifically, CemrgApp provides a tool for left atrial anatomical and structural cardiac MRI analysis, which measures an anatomy and estimates tissue fibrosis burden. Multiple studies have been performed by us and others, which confirm the excellent inter and intra-observer variability of the atrial image analysis workflow. We have used this workflow in reproducibility assessment of atrial fibrosis [13], evaluation of left atrial scar formation [14], and optimisation of LGE-CMR imaging of post-ablation atrial scar [15]. Independent image analysis studies have used the tool to verify that CemrgApp’s atrial fibrosis analysis replicates the results from a third-party software [16], [17]. Furthermore, CemrgApp atrial scar analysis has been demonstrated in scans from different vendors (Siemens and Phillips) and validated with public image data bases [18]. The output of the scar quantification tool for left atrial image analysis is a surface mesh that can be analysed, imported into electro-anatomical mapping system, or used as the basis for creating a patient-specific model. The mesh with the estimated fibrosis burden can then be augmented with a universal coordinate system and atrial fibres [19], derived from an atlas of ex-vivo human diffusion tensor MRI (DTMRI) [20]. The fibrosis burden can be used to estimate tissue conduction and cellular electrophysiology properties to generate a patient-specific model. We have previously used this approach to generate cohorts of patient-specific models [10]. However, the current available tools for creating these models were often in different software platforms, taking on average 4.5 h of processing time per patient case [21].

Despite the extensive research done in the area of patient-specific cardiac models, a notable gap in the literature remains regarding reproducibility studies that address intra and inter-observer variability. This gap is important because accounting for uncertainty in model predictions due to operator variability is crucial for ensuring confidence in simulation predictions in clinical and regulatory applications. Furthermore, bridging the gap would impact existing modelling approaches’ accuracy whether these models guide procedures [22], [23], or their outputs are used as inputs to classifiers for predicting clinical outcomes [10]. In this context, we **aim** to fill this gap by (i) introducing an open-source workflow for creating patient-specific atrial models from cardiac MRI and (ii) performing an intra and inter-observer study to demonstrate the reproducibility in the approach for creating patient-specific left atrial models and quantify uncertainty in model predictions introduced by manual steps in model creation. The **objective** of this study is to quantify the impact of intra and inter-observer variability on atrial fibre maps, activation simulations, and fibrillation simulations. We demonstrate that with a guided, semi-automated modelling approach we can generate operator-independent patient-specific left atrial models. By providing the first evaluation of model reproducibility, we can provide estimates of the degree of uncertainty due to manual operations that give context for interpreting clinical and research simulation studies. Section 2 describes the data and refers to the image acquisition protocols, as well as the users, assignment of cases, and training resources developed. Section 3 describes the methodology, simulation protocols, and the reproducibility experiments and metrics evaluated. Section 4 presents the results of all the experiments (see Fig. 1).

## 2 Materials

**Fig. 2:**
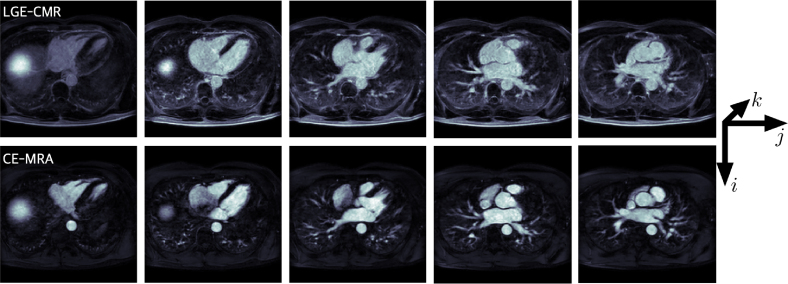
Example axial slices of input volumes. Each dataset consisted of an LGE-CMR scan (top) and its corresponding CE-MRA scan (bottom). Scans were screened to ensure the orientation is RAI (Right-to-left, Anterior-to-posterior, Inferior-to-superior). Scans were resampled to be isotropic, that is, each voxel had a resolution of $1\;{\mathrm{mm}}^{3}$.

Fifty cases were analysed, each consisting of two scans: an ECG-triggered, contrast enhanced magnetic resonance angiogram (CE-MRA), and late gadolinium enhanced cardiac magnetic resonance (LGE-CMR). CMR imaging was performed on Phillips and Siemens 1.5T scanners [13]. The full description of image acquisition is reported by [15]. All input DICOM files were resampled to be isotropic, reoriented and stored in the Nifti-2 format, which ensures data anonymisation. Images were resampled so each voxel had a resolution of $1\;{\mathrm{mm}}^{3}$. All images were screened to fit in the *RAI* orientation, which orients the voxels in the X axis from Right-to-left, the Y axis from Anterior-to-posterior, and the Z axis from Inferior-to-superior (Fig. 2).

### Data allocation and training.

Data were divided amongst five users: the developer (d), three novice users (abc), and one senior user with more experience with medical images (s). Only the developer had seen and screened each of the cases before they were randomly assigned to each users. Each user (abcd) was randomly assigned 20 cases to process. The data assigned to each user were categorised as follows: (i) **10 cases** for **intra**-operator variability; (ii) **5 cases** for **inter**-operator variability vs. another user (abcd); (iii) **5 cases** for inter-operator variability vs. senior user (s). To quantify inter-operator variability, each of the thirty cases was assigned twice. For the intra-operator variability cases, each user had 5 cases independently repeated from their own set. The developer obtained full knowledge of which cases were repeated in each user’s pool only after model creation and analyses were performed. Users received training resources such as videos of each stage of the data processing. A standard operating procedure (SOP) document was written describing the whole pipeline. Training and documentation are available as Supplementary Material.

## 3 Methods

This study consisted of three stages of processing: an image-to-mesh analysis pipeline, fibre mapping through universal atrial coordinates, and electrophysiology simulations. The software pipeline and the reproducibility assessment of inter and intra-operator variability along each of the three stages are the main contributions of this work.

The image-to-mesh analysis pipeline and fibre mapping were integrated into a single software workflow, which was developed in the CemrgApp framework ([12]), an open-source platform to develop image analysis and computer vision workflows. CemrgApp constitutes a platform used to develop standalone pipelines with a specific task. Standalone pipelines are developed as a sequenced set of buttons called plugins.

For this study, a plugin was developed to streamline the creation of simulation-ready meshes from a CMR scan. The plugin developed involves three processing stages: (1) conversion from a pair of scans to a labelled mesh, (2) calculation of universal atrial coordinates through a docker container (hosted at https://hub.docker.com/repository/docker/cemrg/uac), and (3) mapping DTMRI fibres from an atlas, as reported by [20]. The outputs from the developed pipeline are: the Universal Atrial Coordinates, a labelled mesh with fibres, and a mesh with the fibrosis projection. Each user submitted their cases, which were screened for quality control. The next stage in data processing is to run simulations in openCARP, an open-source simulation environment for cardiac electrophysiology [24].

**Fig. 3:**
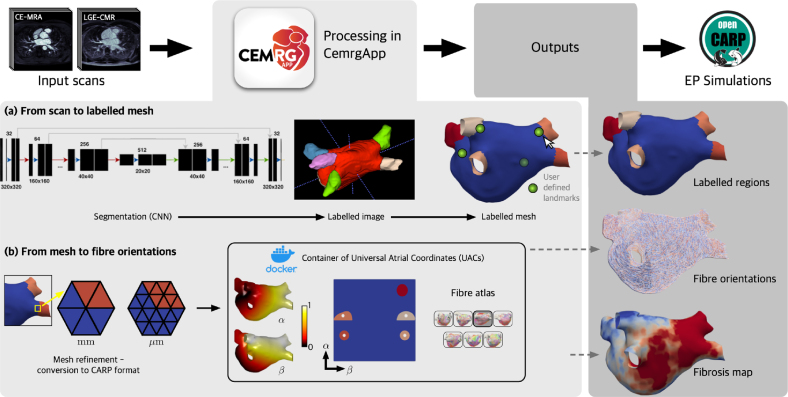
Overview of methodology to process a single MRA/LGE pair. Scans are processed in CemrgApp through a combination of embedded and external code called through docker containers. The pipeline processes the scans (a) from segmentation to a labelled mesh, which is then (b) refined using meshtool ([25]) and processed with the Universal Atrial Coordinates (UAC) docker container. The UAC docker container creates a standardised frame of reference for the mesh, and projects DTMRI fibres from an atlas onto the mesh. Finally, the user produces a fibrosis map from the LGE signal intensity. Outputs produced per case are: a labelled mesh, files with fibre orientations, and a fibrosis map. Electrophysiological simulations are then run on openCARP.

### 3.1 Processing on CemrgApp

This stage processes an input pair of scans, one optimised for atrial anatomy (CE-MRA), one optimised for tissue characterisation (LGE-CMR), and outputs a labelled mesh. A segmentation is produced from the input CE-MRA scan. The segmentation is then registered to the LGE-CMR space, to interrogate the fibrosis score of the left atrium. Labelling of the segmentation is achieved by identifying the pulmonary veins (PVs) and left atrial appendage (LAA). From the labelled segmentation, a corresponding labelled surface mesh is produced. Two modes of operation are available to the user in this stage of the pipeline: *semi-automatic* and *manual*, described below.

#### Semi-automatic pipeline.

A multilabel segmentation of the atrium bloodpool is created using a convolutional neural network (CNN) of the left atrium Razeghi et al. [18]. The multilabel segmentation produces three distinct labels: the left atrial (LA) body, pulmonary veins (PV) and left atrial appendage (LAA), and mitral valve (MV). Naive labels are assigned to each of the pulmonary veins and left atrial appendage to differentiate them. Labels at this stage are assigned from the largest object to the smallest, thus these must later be identified by the user. The surface mesh is generated and the naive labels projected onto it.

#### Manual pipeline.

The user segments the bloodpool of the CE-MRA manually in one of two ways: using the single-label option of the CNN referenced before, or using CemrgApp’s fully manual segmentation module. The single-label option of the CNN ignores the PV/LAA and MV labels created. To standardise how the pulmonary veins and appendage are labelled, the user then identifies the pulmonary veins/appendage by setting control points at the distal end of each vein/appendage. Each point set selection prompts the user to identify the atrial structure they clicked: left atrial body (LA), left atrial appendage (LAA), and pulmonary veins left/right superior/inferior (LSPV, LIPV, RSPV, RIPV). A line is drawn from each point to the centre of mass of the atrium, where the radius of each PV or LAA is calculated and the inflection point of the radius is used to identify the transition of each atrial structure to the atrial body. The location is identified by a disk, which the user can move if necessary. Once accepted, the veins/appendage are labelled according to the user’s selections using default label values.

#### Refining mesh to be simulation-ready.

The following steps are the same for either mode of operation (whether manual or automatic). Meshes at this stage should present six different labels: body (LA), appendage (LAA), and four pulmonary veins (LSPV, LIPV, RSPV, RIPV). The user has the option to manually correct errors in the labelling of the surface mesh. Furthermore, a label verification tool was developed to provide the user with the option to automatically check and correct connectivity issues, for example, if some elements of the atrial body presented the label of the left atrial appendage. The resulting mesh needs to be open at the pulmonary veins and mitral valve. The user clips the mesh by choosing the centres and radii of spheres, which will clip the mesh at the distal ends of the pulmonary vein and at the mitral valve. Once clipped, the mesh is refined to an average edge length of 0.3 mm; then it is cleaned from bad topology definitions, scaled to be in μm, and converted to openCARP format using meshtool [25].

#### Fibrosis map.

The LGE-CMR scan is interrogated by projecting the maximum intensity of the wall onto the surface elements of the clipped mesh. The segmentation was done initially on the CE-MRA scan and registered to the LGE-CMR scan. The wall intensities are estimated by superimposing the surface mesh on the scan and calculate the maximum intensity projection of the voxels along the normal direction of each element. This creates a fibrosis map, to which a threshold can be applied, to find areas with fibrosis or ablation scar. A common technique to determine the threshold is the image-intensity ratio (IIR) [26], in which the threshold is defined by the mean intensity of the bloodpool multiplied by a factor. Common threshold values are 0.97 [13], 1.2, and 1.32 [27]. The pipeline to obtain a fibrosis score has been extensively described before. The reader is referred to the works by [12], [13], [15], [18] for more detailed descriptions.

### 3.2 Universal atrial coordinates in CermgApp

Universal atrial coordinates (UAC) [19], [20] constitute a 2-dimensional frame of reference to compare different atrial geometries. UAC are calculated by solving two Laplace equations with Dirichlet boundary conditions defined by lines in the surface mesh of boundary nodes. The coordinates, (α,β), are defined relative to the atrial structures: pulmonary veins and left atrial appendage. The first coordinate, α, spans from the septal to the lateral walls; the second coordinate, β, is defined from the posterior mitral valve, over the roof to the anterior mitral valve. Once the UAC process is finished, the selected atlas fibre files are mapped onto the specific mesh. The UAC software was packaged into a docker container [28], and ran from within CemrgApp through its command line interface tool. The container can be found through Docker’s repository site at the following link https://hub.docker.com/repository/docker/cemrg/uac.

#### User-selected landmark points.

The user interface of the pipeline displays an interactive view of the mesh where the user selects the landmark points necessary for the calculation of the UAC. Compared to previous implementations, the user is only required to select four landmark points: (1) at the junction between the left superior PV (LSPV) and the atrial body, (2) at the junction between the right superior PV (RSPV) and the atrial body, (3) on the lateral wall, between the LSPV, MV and LAA and (4) on the septal wall, near the *fossa ovalis* (FO). The points are visible in Fig. 3. Having less points selected reduces user input error in the UAC software.

#### Fibre mapping.

The user chooses which fibre orientations to project onto the mesh: epicardium, endocardium or both (bilayer). The user can also select which fibre field from the atlas reported by [20]. There are seven fibre files (1,…,7), an average fibre field, (a), which aggregates the fibre orientations of all cases, and a rule-based fibre field (l) by [29]. For this study, two fibre fields were mapped onto the processed meshes, corresponding to DTMRI fibre file 1 and the Labarthe fibre file.

### 3.3 Reproducibility experiments from CemrgApp pipeline

Reproducibility calculations were evaluated to assess variability of output from the CemrgApp pipeline. Operator variability is assessed **between** (inter) users and **within** (intra) the same user. Evaluations consisted of: shape measurements, fibrosis agreement, and fibre orientation agreement.

#### Shape measurements.

The minimum euclidean distance from any point in one mesh to the other was calculated. Three measurements were made on the resulting array of minimal distances: (i) Hausdorff distance [30], defined as the maximum of the array of minimal distances as a *worst-case* scenario; (ii) the mean of the minimal distance; and (iii) the median.

#### Fibrosis agreement.

This metric is assessed on the scar tissue defined by the surface where the fibrosis signal, projected from the LGE-CMR, is above a pre-determined threshold. Fibrosis agreement is assessed through the intra-class correlation coefficient (ICC) [31], [32], which assesses the reliability of ratings by comparing the variability of different ratings of the same subject to the total variation across all ratings and all subjects. There are 6 different ways of calculating the ICC [31]. The variant of ICC calculated in this work is the average raters with absolute agreement.

#### Fibre orientation agreement.

The comparison is done between the fibre orientations corresponding to the closest elements between meshes. The measurement to compare is the absolute value of the dot product between fibre orientations. Note the direction of the fibres only needs to be co-lineal, that is, angles between fibres of 0° or 180° are considered perfectly aligned. The proportion of angle errors below π/8(=22.5°) was calculated as a measurement of fibre agreement [20].

### 3.4 Simulations

Two types of simulations were run on each of the 100 processed cases: baseline pacing to calculate local activation time (LAT) maps and atrial fibrillation simulations for which phase singularity (PS) maps were calculated. The openCARP simulator [24] was used to run the simulations, using the Courtemanche human atrial model [33] with AF electrical remodelling [34]. Similar to the work by [20], longitudinal conductivity was set to 0.4 S/m and transverse conductivity to 0.1 S/m. For baseline pacing, the model was stimulated at the RSPV rim and run for 1 s. Local activation time (LAT) maps were calculated for bilayer model simulations with two of the fibre field 1 and the Labarthe [29] fibre field. AF was initiated using an arrangement of four Archimedean spirals, and phase singularity maps were calculated following our previous study [35].

#### Assessment metrics.

The local activation time (LAT) maps were compared in two ways: first, the pairwise correlation coefficient was calculated from the mapping data, as reported by [36]; then the total activation time was compared in both inter and intra-operator variability cases. The local conduction velocity (CV) was calculated as the inverse of the magnitude of the gradient (1/‖∇LAT‖) [37]. The mean conduction velocity was compared in both inter and intra-operator variability cases. Finally, as reported by [38], two calculations were performed on each pair of phase singularity (PS) maps: the Pearson correlation coefficient and the structural similarity index.

## 4 Results

Five users processed 100 cases in total, where 40 correspond to intra-observer variability and 60 to inter-observer variability. After processing, users submitted their cases for assessment, which were screened for quality control. All meshes are available on Zenodo [39] at the link: https://doi.org/10.5281/zenodo.7433015. Two cases presented a substantial user-error problem: (i) the user identified the right pulmonary veins in incorrect order during the mesh preprocessing stage of the *semi-automatic pipeline*; (ii) another user forgot to clip the mesh’s pulmonary veins before calculating the scar projection. More details can be found in the Supplementary Material.

### Point/element correspondence for comparisons.

We created mappings between closest points and closest elements from meshes being compared and the distance between these. We eliminated points farther than 1 mm apart from each other. Thus, every comparison apart from the shape agreement measurements is between points (or elements) closer than 1 mm.

### 4.1 Reproducibility from the CemrgApp pipeline

The mean and median times to complete the whole pipeline were 26 min and 25 s and 16 min 43 s, respectively. Most cases were completed between 14 and 36 min. Four outliers were identified, where the process took 52, 100, 148, and 232 min respectively. The hardware used varied from a laptop with 8 GB RAM and 4 cores to a desktop workstation with 128 GB RAM and 64 cores. In nine cases the time logging document was not created or deleted by the user. Users attempted the *semi-automatic* pipeline (Section 3.1) and only defaulted to the *manual* pipeline when the multi-label segmentation presented problems. Problems with the automatic segmentation were primarily related to the segmentation or labelling of the pulmonary veins. For example, the neural network would output a good atrial body geometry, but presented missing or joined pulmonary veins which required manual intervention. The distribution between semi-automatic and manual cases -out of 100- was 51 and 49, respectively. None of the manual cases required the user to perform a fully manual segmentation.

#### Shape measurements.

**Fig. 4:**
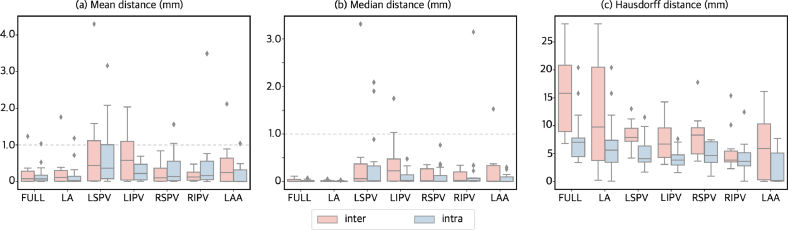
Distance to closest point boxplots of the different metrics: (a) mean, (b) median, and (c) Hausdorff distance. Inter and intra-operator variability plots are shown in pink and blue, respectively. Different boxplots are presented to visualise the different structures: left atrial body (LA), left atrial appendage (LAA), as well as the pulmonary veins left superior (LSPV) and inferior (LIPV), and right superior (RSPV) and inferior Mean (a) and median (b) of the distance to closest point are overall under 1 mm.

The calculations of Hausdorff distance, mean and median were calculated per atrial structure, full atrium, left atrial body, left/right superior/inferior pulmonary veins, and left atrial appendage. Given the resolution of the image, an error of 1 mm or less was considered *acceptable*. The majority of the mean and median smallest-distances were below 1 mm, the left superior and inferior pulmonary veins presented the largest differences. Only the left pulmonary veins reached values above 1 mm in the mean distance, although the 75th percentile were still calculated around or under 1 mm. The Hausdorff distance, as a *worst case scenario* measurement, had a mean value of 9.5 mm, but had ranges reaching almost 30 mm in the inter-operator left atrial body due to the uncertainty in the positioning of the mitral valve in the manual pipeline cases. In all measurements, intra-operator variability results were notably smaller compared to the results corresponding to the inter-operator variability. Fig. 4 shows boxplots showing the different measurements.

#### Fibrosis agreement.

**Fig. 5:**
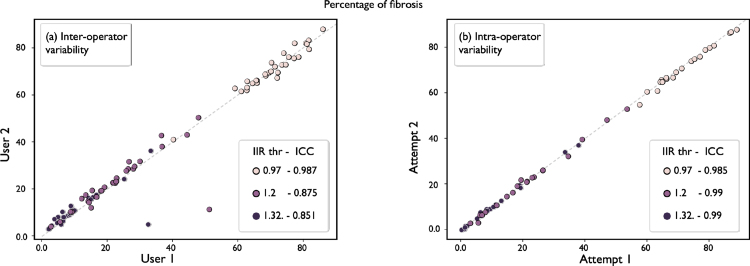
Fibrosis agreement. Inter- (left) and intra-operator (right) variability are presented by showing the different fibrosis scores. On both axes represent the fibrosis score ranging from 0 to 1. Different colours represent different thresholds of the IIR method, which is presented next to the ICC coefficient. Points close to the identity line show good agreement.

Fig. 5 shows the comparisons in fibrosis between and within users. We calculated ICC overall and per IIR threshold. The IIR threshold is calculated by multiplying the mean of the bloodpool by the factor, in case of this study 0.97, 1.2, and 1.32. See Section 3.1 for more details. On **inter-**operator variability comparisons ICC results were: $ICC_{0.97}=0.987$, $ICC_{1.2}=0.875$, $ICC_{1.32}=0.851$ and $ICC_{all}=0.909$. On **intra-**operator variability comparisons ICC results were: $ICC_{0.97}=0.985$, $ICC_{1.2}=0.999$, $ICC_{1.32}=0.999$ and $ICC_{all}=0.999$. We have included a table in the Supplementary Material, which includes a full overview of the ICC coefficients, p-values, and 95% confidence intervals.

#### Fibre orientation agreement.

The absolute value of the dot product, a proxy for the angle between fibre orientations, was calculated in pairs of meshes compared. Elements in corresponding meshes at a greater distance than 1 mm were not added to the calculation, given some of the differences in some meshes, as seen in Fig. 4(c). Results were separated into the different atrial structures and stacked to showcase the distribution of values. Values near 1 represent perfect alignment, as it indicates an angle between fibre orientations of 0°, or 180°. To indicate *good agreement*, a threshold of 22.5° was chosen. In the inter-operator variability pairings, fibres in *good agreement* represented approximately **60.63%** of all the fibres orientations across all cases. In the intra-operator variability pairings, the percentage of fibres in good agreement was approximately **71.77%**.

**Fig. 6:**
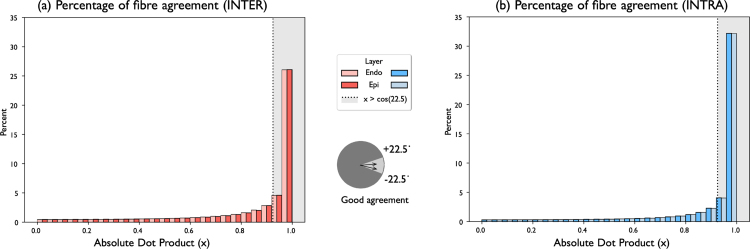
Fibre orientation agreement distribution. (a) Inter- and (b) intra-operator histograms of the distribution of the absolute value of the dot product between two fibres orientations. The histograms corresponding to the different atrial layers (endocardium and epicardium) have been distinguished to show relative distributions. Values greater than cos(22.5°)≈0.924 correspond to angles between fibres between ±22.5°, these were considered in *good agreement*.

### 4.2 Simulation results

**Local activation time (LAT) maps** had an excellent average correlation for both inter and intra comparisons, with mean (± sd) correlations of 0.992 (± 0.007) and 0.996 (± 0.003), respectively. Medians (± IQR) of total activation times for inter and intra calculations were 131.31 (± 18.59) and 132.61 (± 19.08), respectively. The median (± IQR) of the absolute difference of the total activation times was 2.02 (± 2.45) ms for inter, and 1.37 (± 2.45) ms for intra. The Wilcoxon rank test for medians obtained a p-value of 0.86 and 0.83 for inter and intra, which suggests the test could not reject the hypothesis of equal medians in the distributions of total activation times.

The **mean conduction velocity (CV)** was statistically different in almost all cases, where only 10 cases out of 50 (split as 5 in inter and 5 in intra) could not be determined as statistically different. In contrast, the average of the mean conduction velocity (CV) could not be determined as statistically different. The average difference (± sd) of the mean CV was −0.00404 (± 0.0155) m/s for inter, and 0.0021 (± 0.0115) m/s for intra comparisons. The p-values for the comparisons between difference in average mean conduction velocity were found to be 0.535 for inter and 0.771 for intra. Finally, the **phase singularity maps** showed a mean (± sd) correlation of 0.305 (± 0.25) for the inter and 0.248 (± 0.19) for the intra-operator variability comparisons. Regarding the structural similarity index, the mean (± sd) values for inter and intra were higher at 0.648 (± 0.21) and 0.608 (± 0.15), respectively.

**Fig. 7:**
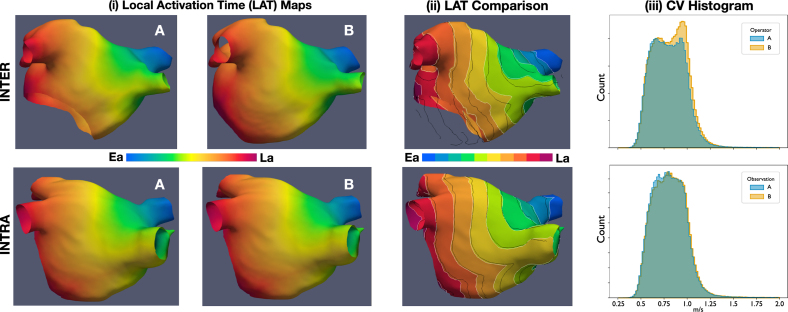
Example of simulated Local Activation Time (LAT) Maps and Conduction Velocity histograms. **Top row**: comparison between operator A vs operator B for inter-operator variability. **Bottom row**: comparison between observation A vs observation B from one of the operators. **Columns**. (i) Local Activation Time Maps shows two observations of the same case, inter or intra depending on the row. (ii) LAT Comparison shows the LAT map from A with white contours, the contours of B’s LAT are superimposed in black. (iii) The distribution of Conduction Velocity (CV) is shown for both A and B. Ea, Early activation; La, Late activation; CV, Conduction Velocity; LAT, Local Activation Time.

Fig. 7 shows two examples of simulation outputs, corresponding for a case of inter and intra-operator variability. Each LAT map is presented with a colourmap ranging from early activation (Ea) to late activation (La). A qualitative comparison between local activation time (LAT) maps is shown in column (iii), where the contours are shown overlapped to appreciate visually the differences in propagation patterns. The histograms corresponding to each observation local conduction velocity are shown overlapped. In the case for inter-operator variability, meshes have a notably different geometry around the mitral valve. This can be appreciated more closely in column (iii) of Fig. 7.

## 5 Discussion

This work describes an extension of CemrgApp to create simulation-ready meshes from a pair of CMR scans. CemrgApp is a software platform designed to be extended through standalone plugins. We presented a model reproducibility study, where 50 cases were distributed amongst five users to generate 100 models of the left atrium with two sets of fibre orientations. Before analysing the reproducibility results, we discuss two general points. First, the processing of cases between semi-automatic and manual cases was 51 to 49, respectively. It is worth noting that the high number of cases where users decided to use the manual workflow was mainly due to labelling errors rather than an incorrect segmentation of the atrial body. In spite of this, the time to process a case was substantially reduced from 4.5 h (15 GB, 4 cores) in the work by [21], to the median value of 16 min and 43 s in this study. Even considering only the use cases where a laptop was used, the time to process a case was reduced to a median value of 17 min and 32 s. It does not appear the hardware constituted a bottleneck in the processing time. Second, the quality control stage carried out after collecting the data from users, where only two instances of user error were found and corrected. It is important to note that the quality control stage described in Section 4 was carried out without introducing bias in the analysis. As discussed in the supplementary material, (i) fixing the labels assigned to the pulmonary veins is an automatic process, which has no impact on the user’s decision on the position or shape of the veins; (ii) clipping the pulmonary veins in the scar projection mesh utilises the user’s pre-defined clippers.

### Shape agreement.

Mean and median distances were within 1–2 mm, which is close to the image resolution for all atrial structures. Left pulmonary veins presented minimum distance values higher than 1 mm in some cases. The main reason was the variability when deciding where the vein starts and where to clip the vein. For the *worst-case-scenario* calculation of the Hausdorff distance, the larger problems were in the left atrial body and appendage, where differences were larger than 5 mm. The mean Hausdorff distance of 9.5 mm was comparable to other segmentation studies, reporting mean Hausdorff distances of 20 mm and 4.2 mm from [40], [41], respectively. It is worth noting that the studies were of fully automatic CNN-based methods. Furthermore, [40] reported a Hausdorff distance of 36.4 mm from a competing U-Net-based segmentation, whilst the maximum Hausdorff distance in this study was of 29.9 mm. The main problem with the left atrial body was the clipping of a mitral valve, which varied substantially. The largest differences were found in the inter-operator variability comparisons.

### Fibrosis agreement.

Compared to the reproducibility measurements by [13], our fibrosis agreement was overall higher at an ICC of 0.909 for inter and an ICC of 0.999 for intra-operator variability. If the thresholds are analysed independently, then the results become comparable to our previous study, with the lowest ICC in the inter-operator variability at an IIR of 1.32. From Fig. 5 (left), two data points stand out, corresponding to measurements at IIR = 1.2 and IIR = 1.32. For context, [13] investigated reproducibility of manually segmenting the atrial body, identifying and clipping of the mitral valve, pulmonary veins and left atrial appendage; intra-class correlation (ICC) coefficients of 0.88 for inter and 0.94 for intra-operator variability were reported.

### Fibre orientation agreement.

In both inter and intra-operator variability tests, the distribution of fibre orientations appear in good agreement. In the distributions presented on Fig. 6, where 1 corresponds to perfect agreement, the percentages of angle between ±22.5° are 60.63% in inter-operator variability cases, and 71.77% in the intra-operator variability cases. Put into context, in [20] reported fibre agreement of approximately 33.36% (±6.88), when comparing between the fibre fields 1 and the rule-based Labarthe field, used also in this work.

### Simulation results.

Our results support that the distribution of total activation time is similar for both inter and intra comparisons. First, the Wilcoxon rank test for total activation time was not statistically significant, indicating that the assumption of equal means could not be rejected for either inter or intra comparisons. Furthermore, the medians and inter-quartile range were very similar for both inter and intra-observer variations. This is comparable to the differences between different atrial fibre fields, reported by [20], which would make inter and intra-observer uncertainty on the scale of inherent uncertainty due to the inability to measure the atrial fibres. A similar case occurred with the mean conduction velocity, although the individual comparisons were significantly different based on the output of each individual t-test. The examples in Fig. 7 show a closer resemblance of the contour lines between early activation (Ea) and late activation (La) in the intra-operator variability outputs. Compared to the inter-operator variability, which shows a notably different shape. This result is consistent with the other agreement metrics shown in this work, such as shape, fibrosis, or fibre agreements. The comparison metrics presented show a low correlation between the PS maps, and only a *modest* similarity index. We note that with increased complexity, the risk of adding in variation increases. Thus, even when local activation times will generate consistent results, fibrillation results are invariably more prone to variation as it constitutes a more complex simulation. In [20], the mean correlation of PS maps between fibre fields ranged low from 0.14–0.44, and results varied based on fibre field and anatomy. It is worth noting that we performed only a single simulation of AF, however aggregating PS maps across multiple pacing protocols [22], [42] may lead to more consistent results. Longer simulation times may also lead to more stable results [43].

### 5.1 Limitations

#### Limitations of the CemrgApp pipeline.

The largest difference in shape came from the positions of the mitral valve, when selected manually. This could be overcome by enhancing the manual variant to keep the mitral valve segmentation and use it, removing user input from it. At the moment this is only possible in the automatic variant of the application. This limitation impacts the generation of the universal atrial coordinates, since they depend on the geometry of the meshes, which in turn affects the fibre mapping and simulation outputs. We are currently developing an extension to the universal atrial coordinates that removes the requirement to label the mesh.

#### Limitations to the universal atrial coordinates implementation.

The Universal Atrial Coordinates pipeline assumes there are four pulmonary veins. For the CemrgApp plugin, we incorporated tools to ignore smaller veins detected. A possible extension could be to mark the location of these extra veins in the universal atrial coordinates, to allow a more detailed investigation in the impact these structures.

## 6 Conclusion

We have presented an open-source, pipeline to produce models of the left atrium starting from a pair of CMR scan(s) through to a simulation-ready mesh with (1) estimated fibrosis, and (2) fibre orientations projected onto the surface of the mesh. We produced 100 models that test inter and intra-operator variability of the pipeline (split 60/40). Although there were notable differences, starting in the shape agreement metrics, which propagated errors down the pipeline, both inter and intra-operator variability was comparable with uncertainty in atrial models due to image resolution or the use of estimated fibre orientations.

### Practical implications.

(i) Patient-specific computational models of the heart are increasingly been used to develop and guide clinical therapies [1]. The software pipeline we have developed aims to support upcoming frameworks for the generation of patient-specific atrial models, which have an impact on the development of personalised therapies for atrial fibrillation and other illnesses. (ii) The uncertainties presented in model anatomy, fibres and simulations provide a context for interpreting of simulation study results. Thus, this work offers the first reproducibility study as a potential initial template for reporting simulation-study reproducibility, thus providing a benchmark for future improvements in model creation. (iii) The software has a low barrier to entry and a low learning curve, making it accessible to a wide range of users. Users adapted to the software pipeline quickly with minimal training consisting of up to an hour session and resources like instructional videos and a standard operating procedure document, available as supplementary material. The software is fully open-source, can be run in a standard laptop computer in a shorter time, and its methodologies are standardised. All of these reasons are important considerations for clinical applications.

### Final remarks.

Overall, we consider this software pipeline to represent a substantial contribution to the development of patient-specific computational models of the heart, which will facilitate the transition towards the adoption of computational models into clinical applications and pave the way for more research, with larger cohorts.

## Access to code, binaries, and documentation

The version of CemrgApp with the plugin developed for this work is hosted on Github under commit number 0539e31, which at the time of writing can be accessed at https://github.com/CemrgAppDevelopers/CemrgApp/tree/0539e31. Binaries for Windows, Linux (Ubuntu) and macOS (intel) can be made available upon request. Besides the standard operating procedure document submitted as supplementary material, tutorial videos have been uploaded to Youtube for the automatic pipeline and manual pipelines at the respective urls https://youtu.be/zU_czEPaCIs, and https://youtu.be/G4G4y-QuVV4.

## CRediT authorship contribution statement

**José Alonso Solís-Lemus:** Conceptualization of this study, Methodology, Software, Data processing, Manuscript. **Tiffany Baptiste:** Software, Data processing. **Rosie Barrows:** Software, Data processing. **Charles Sillett:** Software, Data processing. **Ali Gharaviri:** Software, Data processing. **Giulia Raffaele:** Software, Manuscript. **Orod Razeghi:** Software, Manuscript. **Marina Strocchi:** Software, Manuscript. **Iain Sim:** Data acquisition, Software. **Irum Kotadia:** Data acquisition, Software. **Neil Bodagh:** Data acquisition, Software. **Daniel O’Hare:** Data acquisition. **Mark O’Neill:** Data acquisition. **Steven E. Williams:** Data acquisition, Methodology. **Caroline Roney:** Conceptualization of this study, Simulations, Methodology, Software, Manuscript. **Steven Niederer:** Conceptualization of this study, Methodology.

## Declaration of Competing Interest

The authors declare that they have no known competing financial interests or personal relationships that could have appeared to influence the work reported in this paper.

## Appendix A Supplementary data

MMC S1 Including: (1) Access to code, data, and training materials; (2) detailed description of methods; (3) extended results.
