# Supplementary material for: Evaluation of an open-source pipeline to create patient-specific left atrial models: A reproducibility study
Source: Comput Biol Med. 2023 Aug;162:107009. doi: 10.1016/j.compbiomed.2023.107009 (PMC10790305; doi:10.1016/j.compbiomed.2023.107009)
Supplement: MMC S1 — Including: (1) Access to code, data, and training materials; (2) detailed description of methods; (3) extended results. [file mmc1.pdf]

Highlights

Evaluation of an Open-Source Pipeline to Create Patient-Specific Left Atrial Models: A Reproducibility Study
Supplementary Material

José Alonso Solís-Lemus,Tiffany Baptiste,Rosie Barrows,Charles Sillett,Ali Gharaviri,Giulia Raffaele,Orod Razeghi,Marina Strocchi,Iain Sim,Irum Kotadia,Daniel O'Hare,Mark O'Neill,Steven E Williams,Caroline Roney,Steven Niederer

- New open-source pipeline creates patient-specific left atrial models from CMR scans.
- Inter/intra-operator variability evaluated in study with 100 models.
- Model building time reduced to just 16.7 minutes.
- Error measurements from operator variability comparable to image resolution/fibre estimation.

Evaluation of an Open-Source Pipeline to Create Patient-Specific Left Atrial Models: A Reproducibility Study Supplementary Material

Dr José Alonso Solís-Lemus[a,*,1], Tiffany Baptiste[a], Rosie Barrows[a], Charles Sillett[a], Dr Ali Gharaviri[a,c], Giulia Raffaele[a], Dr Orod Razeghi[d,a], Dr Marina Strocchi[a], Dr Iain Sim[a], Dr Irum Kotadia[a], Dr Daniel O'Hare[a], Prof Mark O'Neill[a], Dr Steven E Williams[a,c], Dr Caroline Roney[a,b,f] and Prof Steven Niederer[a,e,f]

[a]Biomedical Engineering, King's College London, St Thomas Hospital, , London, SE1 7EH, , UK
[b]Queen Mary University of London, Mile End Rd, Bethnal Green, London, E1 4NS, , UK
[c]Centre for Cardiovascular Science, University of Edinburgh, Old College, South Bridge, Edinburgh, EH8 9YL, , Scotland
[d]Department of Haematology, NHS Blood and Transplant Centre, University of Cambridge, Cambridge, , , UK
[e]Alan Turing Institute, British Library, 96 Euston Rd, London, NW1 2DB, , UK
[f]Both collaborators serving as joint last-authors,

ARTICLE INFO

Keywords:
Patient-specific Modelling
Reproducibility
Cardiac Electrophysiology
Atrial Imaging
Image Analysis
Digital Twins

ABSTRACT

This is the supplementary material document for the manuscript entitled Evaluation of an Open-Source Pipeline to Create Patient-Specific Left Atrial Models: A Reproducibility Study. This paper presents an open-source software pipeline to create patient-specific left atrial models with fibre orientations and a fibrosis map, suitable for electrophysiological simulations. The semi-automatic pipeline takes as input a contrast enhanced magnetic resonance angiogram, and a late gadolinium enhanced (LGE) contrast magnetic resonance (CMR). Five operators were allocated 20 cases each from a set of 50 CMR datasets to create a total of 100 models to evaluate inter and intra-operator variability. Each output model consisted of: (1) a labelled surface mesh open at the pulmonary veins and mitral valve, (2) fibre orientations mapped from a diffusion tensor MRI (DTMRI) atlas, (3) fibrosis map extracted from the LGE-CMR scan, and (4) simulation of local activation time (LAT) and phase singularity (PS) mapping.

A. Access to Code and Binaries

The version of CemrgApp with the plugin developed for this work is hosted on Github under commit number `0539e31`, which at the time of writing can be accessed at `https://github.com/CemrgAppDevelopers/CemrgApp/tree/0539e31`. Binaries for Windows, Linux (Ubuntu) and macOS (intel) can be made available upon request.

B. Access to Data and Training Materials

Models have been made available on Zenodo under the DOI `10.5281/zenodo.7433015`. Besides the standard operating procedure document submitted as supplementary material, tutorial videos have been uploaded to Youtube for the automatic pipeline and manual pipelines at the respective urls `https://youtu.be/zU_czEPaCIs`, and `https://youtu.be/G4G4y-QuVV4`.

*Corresponding author
`jsolislemus@kcl.ac.uk` (J.A. Solís-Lemus)
`alonsojasl.github.io` (J.A. Solís-Lemus)
ORCID(s): 0000-0002-2596-7811 (J.A. Solís-Lemus); 0000-0003-3157-3568 (T. Baptiste); 0000-0001-7155-9483 (R. Barrows); 0000-0003-2930-7818 (C. Sillett); 0000-0003-1803-8237 (A. Gharaviri); 0000-0003-0925-4632 (G. Raffaele); 0000-0003-4989-5565 (O. Razeghi); 0000-0003-1312-251X (M. Strocchi); 0000-0002-7266-2084 (S.E. Williams); 0000-0001-6809-0928 (C. Roney); 00000-0002-4612-6982 (S. Niederer)

C. Allocation and distribution of cases per user

Data were divided amongst five users: the developer (d), three novice users (abc), and one senior user with more experience with medical images (s). Only the developer had seen and screened each of the cases before they were randomly assigned to each user. The allocation and distribution of cases per user and role of senior user are presented in Figure 1, where an example of data allocation is presented for a pair of users (from the abcd). The role of the senior user (s) is shown on the right hand side of the Figure. Each user (abcd) was assigned randomly 20 cases to process. The data assigned to each user were categorised as follows: (i) **10 cases** for **intra**-operator variability; (ii) **5 cases** for **inter**-operator variability vs. another user (abcd); and (iii) **5 cases** for inter-operator variability vs. senior user (s).

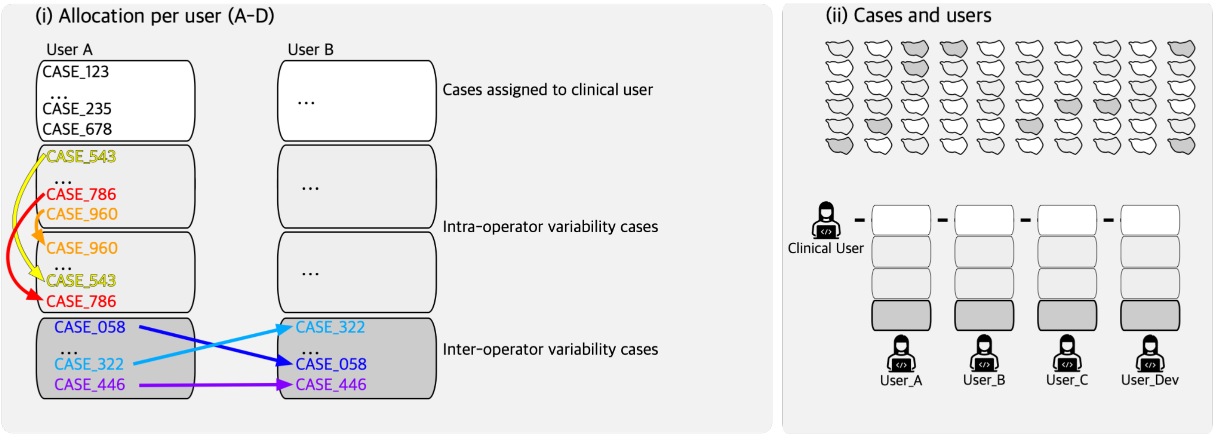

Figure 1: Allocation and distribution of cases per user and role of senior user. (a) Shows the allocation of cases per pair of users. Grayscale represents the category of case allocated. (b) Shows the distribution of case category from the pool of fifty. Twenty out of 50 were non-repeated cases (white), 20 out of 50 were used for intra-operator variability, and 10 out of 50 were used for inter-operator variability (dark grey).

D. Methods

The CemrgApp plugin developed for this work is based on a series of buttons. Figure 5 show a graphical representation of the buttons and their conncetion to Sections 3.1 and 3.2 of the main text.

D.1. Semi-automatic vs manual pipelines

The CemrgApp plugin presented introduced two modes of operation to produce a labelled mesh: *semi-automatic* and *manual*. the semi-automatic pipeline is based on the work by Razeghi et al. (2020), which was trained on 207 cases achieving a dice score of 0.91 ± 0.2. Figure 2 shows a graphical representation of the way both pipelines integrate seamlessly into a mesh with labelled elements representing the different atrial structures. The diagram also shows the point where the MRA scan is registered with the LGE to later perform the fibrosis identification. Finally, the creation of a simulation-ready mesh is done by clipping the mesh at the distal ends of the pulmonary veins and the mitral valve. Then, the mesh is refined to an edge length of 0.3 mm and scaled to be in μm.

D.2. Mesh post-processing and refining

Tools were developed as a part of the CemrgApp pipeline presented to manually edit labels and to automatically correct errors in labelling, see Figure 3. The users can manually edit labels by setting control points over the desired areas in the surface mesh, then they select the label they wish to use to cover. The user then presses a button, which will define the shortest geodesic paths between the points, in the order they were selecter, and assign the selected label onto all the points in the corridor and their neighbouring elements in the mesh. An automatic verification tool was also developed, which iterates through the labels in the mesh, checking there are no *islands* of elements with a different label than what they should be. For example, in Figure 3 (centre), mislabelled elements are highlighted. These would be re-labelled automatically to the correct value.

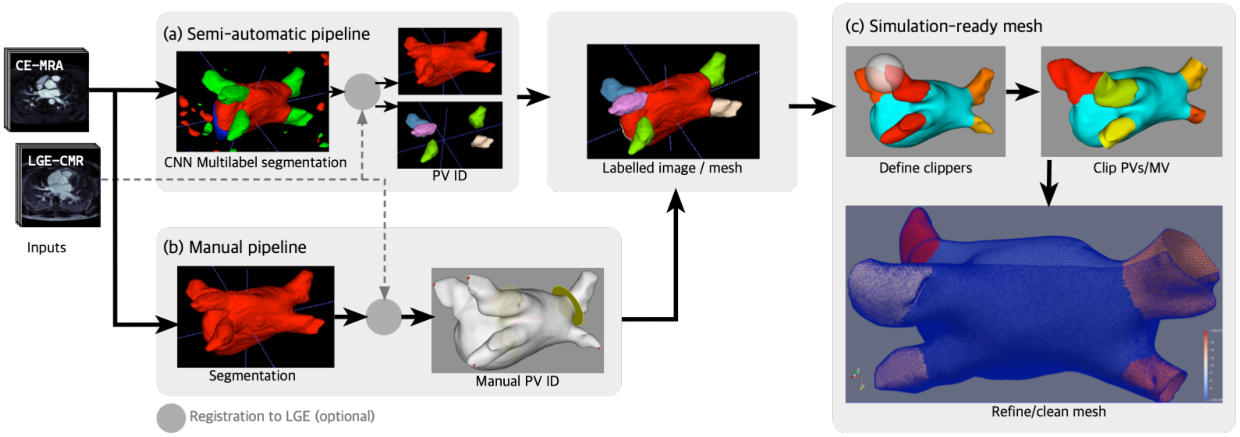

Figure 2: Distinction between semi-automatic and manual segmentation and pulmonary vein identification in CemrgApp module. From left to right, the input scans (MRA and LGE) are loaded into the system and segmented with a multi-label CNN-based automatic segmentation (a) or a manual segmentation (b). The automatic segmentation is from the work by Razeghi et al. (2020), it has three labels: atrial body, pulmonary veins, and mitral valve. The manual segmentation can be done by editing the single-label version of the CNN referenced before. The dotted arrow shows the moment in the pipeline where the segmentation –based on the MRA scan– is registered to the LGE scan. To create a simulation-ready mesh (c), the pulmonary veins are clipped by user-defined spheres at the distal end of the pulmonary veins. The mitral valve is also removed. Finally, the mesh is refined to an edge length of 0.3mm and rescaled to be in μm.

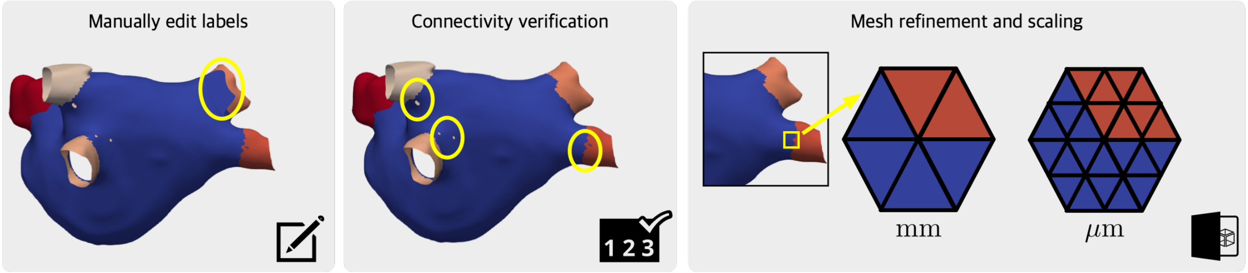

Figure 3: Mesh post-processing and refining. Tools for manual editing (left) and automatic error-correction (centre) of labels in meshes are incorporated in the CemrgApp pipeline. Finally, mesh refining and cleaning (right) is done through meshtool, by Neic et al. (2020), which is incorporated in CemrgApp through openCARP's docker container.

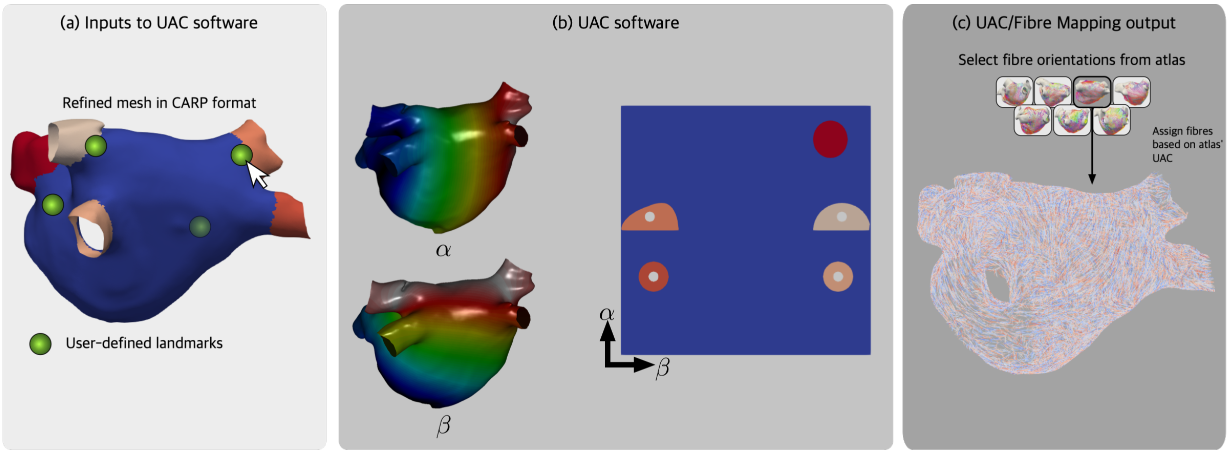

Figure 4: Universal Atrial Coordinates and Fibre Mapping Stages. The diagram shows the steps from the user landmarks to the fibre mapping.

D.3. Connection between CemrgApp and the Universal Atrial Coordinates docker container

The user selects four landmark points, which guide the creation of the Universal Atrial Coordinates (UAC) Roney et al. (2019). The UAC code has been packaged in a docker container (`hub.docker.com/repository/docker/cemrg/uac`). The universal atrial coordinates allow the mapping of fibre orientations from an atlas Roney et al. (2021). Detailed description of the UAC and fibre mapping algorithm are found in the works Roney et al. (2019, 2021). Figure 4 shows a diagram of the process from the user landmarks, universal atrial coordinates, and fibre mapping on a particular case.

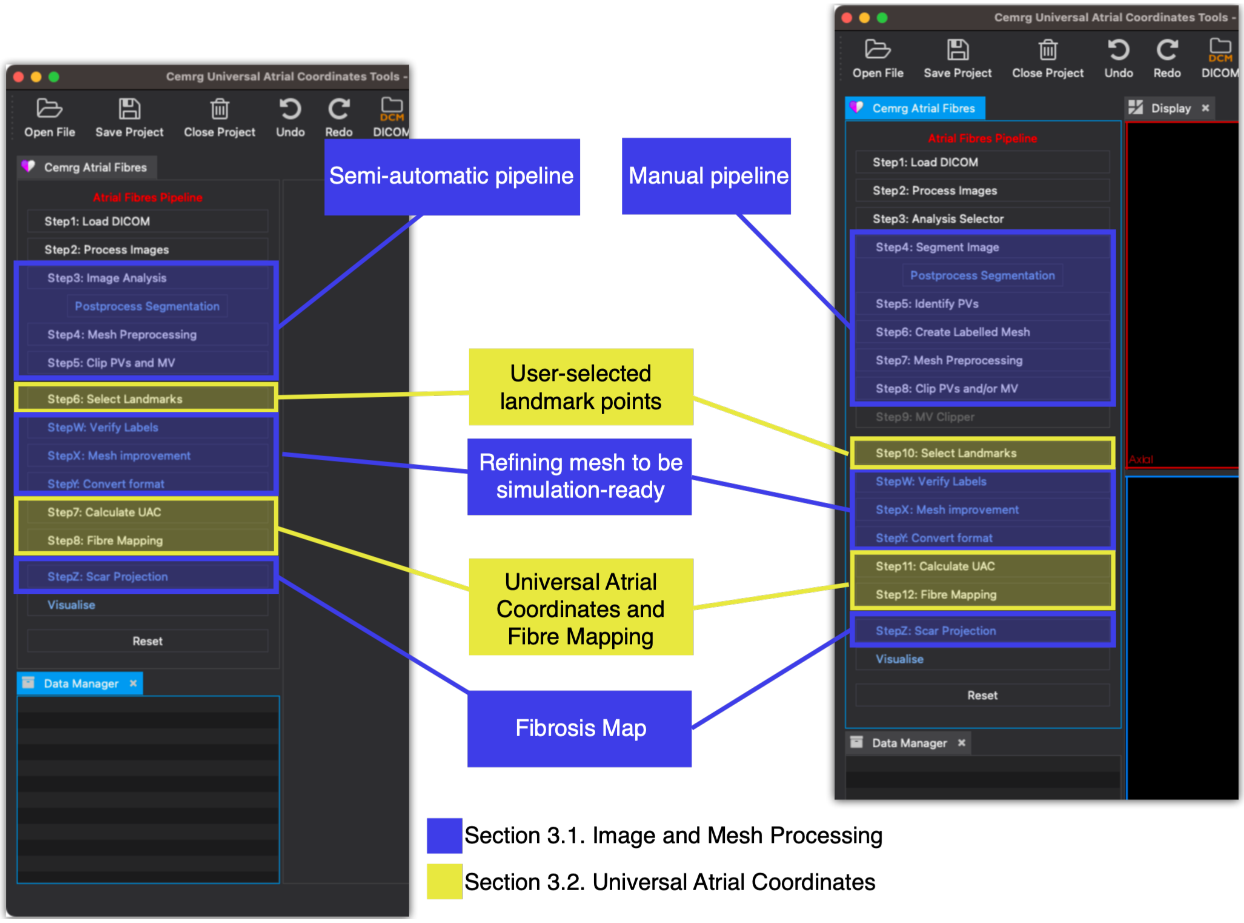

Figure 5: Representation of user interface (UI) elements in the CemrgApp plugin and their connection to the description in the main text. The UI elements are grouped into two categories: (1) buttons to do Image and Mesh Processing (blue), and (2) buttons to perform the Universal Atrial Coordinates (UAC) and Fibre Mapping processing (yellow). Two instances of the UI are shown as some of the buttons change depending on the processing mode selected by the user. Left: semi-automatic processing mode. Right: manual processing mode. Notice that the manual pipeline show more buttons, as the steps require more user input. Buttons are numbered automatically depending on the processing mode selected.

E. Results

E.1. Quality control

The 100 cases processed were screened for quality control. Two cases presented a user problem which, if not addressed, would have exclude them from the analysis. Both cases were from the intra-observer variability sets. The first, where the user identified the RSPV and RIPV in different order during the mesh preprocessing stage of the *semi-automatic pipeline* described in Section 3.1. The case was reprocessed by the same user from the starting pair of scans. The second error was caused when another user calculated the Scar Projection of the pipeline before clipping

Table 1
Intra-class correlation (ICC) coefficient for inter and intra-observer tests in fibrosis score. ICC was calculated overall, and per each IIR threshold 0.97, 1.2, and 1.32. p-values and confidence intervals at 95% are reported.

**Inter**	ICC	pval	CI95%	**Intra**	ICC	pval	CI95%
Overall	0.909	1.77e-9	[0.81 0.96]	Overall	0.999	2.23e-6	[0.99 1.]
0.97	0.987	3.56e-21	[0.97 0.99]	0.97	0.985	6.89e-4	[0.85 1.]
1.2	0.875	9.36e-8	[0.74 0.94]	1.2	0.999	3.03e-7	[0.99 1.]
1.32	0.851	7.06e-7	[0.69 0.93]	1.32	0.999	7.83e-7	[0.99 1.]

the mesh. The surface mesh with the scar projection was clipped using the same clippers saved from the corresponding stage in the pipeline. The fix was performed by the developer, as the user's involvement was already stored in the clipper positions and radii. Figure 6 shows the two cases identified during the quality control screening for reprocessing.

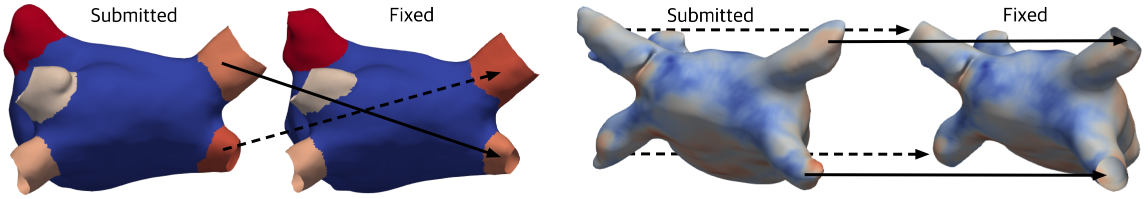

(a) Incorrect labelling

(b) Fibrosis calculation before clipping

Figure 6: Cases identified during the Quality Control stage of this study. In both instances, the submitted and the fixed mesh are indicated with labels and arrows (a) The incorrect labelling shows the right superior and inferior pulmonary veins in incorrect order. In (b), the mesh has not been clipped at the moment of calculating the scar projection.

E.2. Fibrosis Agreement

Table 1 shows the detailed intra-class correlation (ICC) coefficient results in detail for the fibrosis agreement reproducibility tests. ICC was calculated specific to each IIR threshold calculated. Confidence intervalsand p-values are reported. In all cases, the p-value of the ICC is lower than the standard 0.05.

References

Neic, A., Gsell, M.A., Karabelas, E., Prassl, A.J., Plank, G., 2020. Automating image-based mesh generation and manipulation tasks in cardiac modeling workflows using meshtool. SoftwareX 11, 100454. doi:https://doi.org/10.1016/j.softx.2020.100454.

Razeghi, O., Sim, I., Roney, C.H., Karim, R., Chubb, H., Whitaker, J., O'Neill, L., Mukherjee, R., Wright, M., O'Neill, M., Williams, S.E., Niederer, S., 2020. Fully Automatic Atrial Fibrosis Assessment Using a Multilabel Convolutional Neural Network. Circulation. Cardiovascular Imaging 13, e011512. doi:10.1161/CIRCIMAGING.120.011512.

Roney, C.H., Bendikas, R., Pashakhanloo, F., Corrado, C., Vigmond, E.J., McVeigh, E.R., Trayanova, N.A., Niederer, S.A., 2021. Constructing a Human Atrial Fibre Atlas. Annals of Biomedical Engineering 49, 233–250. doi:10.1007/s10439-020-02525-w.

Roney, C.H., Pashaei, A., Meo, M., Dubois, R., Boyle, P.M., Trayanova, N.A., Cochet, H., Niederer, S.A., Vigmond, E.J., 2019. Universal atrial coordinates applied to visualisation, registration and construction of patient specific meshes. Medical Image Analysis 55, 65–75. doi:10.1016/j.media.2019.04.004.
